# Fatigue Profiles in Patients with Multiple Sclerosis are Based on Severity of Fatigue and not on Dimensions of Fatigue

**DOI:** 10.1038/s41598-020-61076-1

**Published:** 2020-03-05

**Authors:** Heleen Beckerman, Isaline CJM Eijssen, Jetty van Meeteren, Marion C Verhulsdonck, Vincent de Groot

**Affiliations:** 1 Department of Rehabilitation Medicine, Amsterdam University Medical Centers, Vrije Universiteit, Amsterdam, The Netherlands; 2 Amsterdam Public Health research institute, Amsterdam University Medical Centers, Amsterdam, The Netherlands; 3 MS Center Amsterdam, Amsterdam University Medical Centers, Amsterdam, The Netherlands; 4 Rijndam Rehabilitation, location Erasmus MC University Medical Center, Rotterdam, The Netherlands; 5 Rehabilitation Center St Maartenskliniek, Nijmegen, The Netherlands

**Keywords:** Diseases, Health care

## Abstract

Fatigue related to Multiple Sclerosis (MS) is considered a multidimensional symptom, manifesting in several dimensions such as physical, cognitive, and psychosocial fatigue. This study investigated in 264 patients with severe primary MS-related fatigue (median MS duration 6.8 years, mean age 48.1 years, 75% women) whether subgroups can be distinguished based on these dimensions. Subsequently, we tested whether MS-related fatigue consists of a single common unidimensional factor. Subscale scores on four self-reported fatigue questionnaires, including the Checklist of Individual Strength, the Modified Fatigue Impact Scale, the Fatigue Severity Scale and the SF36 vitality, were used in a cluster analysis to identify patients with similar fatigue characteristics. Next, all 54 items were included in exploratory factor analysis to test unidimensionality. Study results show that in patients with a treatment indication for primary MS-related fatigue, fatigue profiles are based on severity and not on the various dimensions of fatigue. The three profiles found, suggested one underlying fatigue dimension, but this could not be confirmed. Factor analysis of all 54 items resulted in 8 factors, confirming the multidimensional construct of the included fatigue questionnaires.

## Introduction

Multiple sclerosis-related fatigue is a disabling symptom that affects most patients during the neuroprogressive course of the disease^1–4^. MS-related fatigue is a puzzling interplay between multiple genotypic and phenotypic factors^5,6^. Fatigue can be caused either directly by the MS disease process in the central nervous system (i.e. primary fatigue), initiated by inflammatory processes associated with immune activation, demyelination, axonal loss or neuroendocrine disturbance, or indirectly by other problems (i.e. secondary fatigue) such as insomnia, sleep disturbance due to urge incontinence or spasticity, acute infections, thyroid disorders, physical inactivity and deconditioning, or depression. In all cases, a correct differential diagnosis is required for proper clinical decision making.

There are many definitions of fatigue (see Supplementary Information 1 for references regarding Fatigue Definitions) and over 250 ways to measure fatigue^7^. Examples of frequently used definitions of MS-related fatigue include: the reduction in performance following either prolonged or unusual exertion, together with feelings of sensory, motor, cognitive or subjective fatigue^8^; a subjective lack of physical and/or mental energy that is perceived by the individual or caregiver to interfere with usual and desired activities^9^; the perception of decreased mental or physical energy that may restrict routines of daily activities^10^; the failure to initiate and/or sustain attentional tasks (mental or cognitive fatigue) and physical activities (physical fatigue)^5^.

Dittner *et al*.^11^ recognized the wide range of fatigue definitions as a so-called Catch-22 situation: “Before a concept can be measured, it must be defined, and before a definition can be agreed, there must exist an instrument for assessing phenomenology. There is unfortunately no “gold standard” for fatigue, nor is there ever likely to be”. This Catch-22 situation has caused many researchers to struggle with the assessment, understanding, etiology and classification of fatigue within different patient groups. To produce a workable solution, proposals were made to distinguish non-pathological or normal physiological fatigue from pathological fatigue, general from disease-specific fatigue, brief or acute periods of fatigue from chronic fatigue, and central from peripheral fatigue, (i.e., muscle fatigability due to disorders of muscle and neuromuscular junctions)^5,12–15^. Furthermore, recent evidence suggests that perceived fatigue and energy should be investigated separately as they seem to be two independent constructs^16^. The many elements of fatigue in neurological diseases were included in a proposed unified taxonomy for fatigue, including an assessment approach to addressing distinct aspects of fatigue and fatigability in clinical and research settings^6^.

In patients with MS, fatigue is considered a multidimensional symptom, manifesting itself in distinct dimensions such as physical, cognitive, and psychosocial fatigue. Assessing perceived fatigue in patients with MS is complex due to its multidimensional and non-objectively verifiable character. The most commonly used methods in clinical practice and research are self-reported questionnaires, and many valid, reliable, and responsive unidimensional and multidimensional fatigue questionnaires are currently available. See Supplementary Information 2 for references regarding systematic reviews of fatigue instruments.

Fatigue questionnaires vary greatly regarding the factors examined and may include questions on severity or intensity, duration, momentary perceptions, chronic character, dimensions of fatigue (e.g. mental vs. physical), affective meaning and distress (e.g. motivation), impact of fatigue on daily functioning, behavioural interference with activities and ratings of related constructs (e.g. tiredness or sleepiness) (Supplementary Information 2). As in other diagnostic areas, there is currently no consensus regarding the ideal core set of fatigue questions needed to study MS-related fatigue^17–20^.

In MS rehabilitation and research, multidimensional fatigue scales are most often used. However, scores on subscales of multidimensional fatigue questionnaires are often presented separately, without showing their mutual relationship in individual patients. On the other hand, the presentation of total scores on multidimensional scales results in the loss of information on the underlying individual dimensions^21^.

Developing fatigue profiles might help reduce the number of measurement instruments, and might facilitate decision making on which instruments should be used and which qualify as redundant. More importantly, a patient’s fatigue profile might facilitate clinical decision making with regard to the content and specificity of treatment. Indeed, a clear distinction between different fatigue profiles that show the involvement of a certain domain (e.g. cognitive fatigue or physical activity-induced fatigue) would create opportunities for patient-tailored fatigue treatments. Differentiation between fatigue profiles might also help to detect specific treatment effects and to explain the underlying mechanisms of treatment interventions such as aerobic training, energy conservation management and cognitive behavioural therapy^22^.

The objectives of this study were twofold: (1) to investigate whether in patients with a treatment indication for severe primary MS-related fatigue, fatigue profiles are based on the various dimensions of fatigue, and (2) to test whether there is a single common unidimensional factor model of perceived fatigue in patients with MS.

## Methods

### Design

Baseline data on 264 participants from the TREFAMS-ACE trials were analysed. TREFAMS is an acronym for the TReating FAtigue in MS programme, and ACE refers to the rehabilitation treatment methods under study, specifically Aerobic training, Cognitive Behavioural Therapy, and Energy Conservation Management^22^. The TREFAMS-ACE research programme included three multicentre randomised clinical trials (RCTs) and one explanatory study on the biological mechanisms that underlie MS-related fatigue in general and the treatment effects in particular. The patient enrolment process consisted of a clinical visit in which a rehabilitation physician checked the inclusion and exclusion criteria. After inclusion, self-reported questionnaires were offered to participants via internet or on paper and were completed at home. Filling in the study questionnaires took about 90 minutes. Patients were advised to filling in the questionnaires with some breaks within 1–2 days. The completion had to be ready before randomization to the trial interventions. The list of measurement instruments used in the TREFAMS-ACE studies can be found in our Trials paper of 2013^22^.

### Patients

Enrolment criteria were designed to include severely primarily fatigued MS patients who all had a treatment indication because of their severe fatigue symptoms, but exclude secondary fatigue. Patients had to fulfil the following inclusion criteria: (a) definitive diagnosis of MS, (b) severely fatigued (i.e. a score on the Checklist of Individual Strengths (CIS20r) fatigue subscale ≥ 35), (c) ambulatory patients, (d) no evident signs of an MS exacerbation or corticosteroid treatment in the past 3 months, (e) no current infections, (f) no anaemia, and (g) normal thyroid function. The exclusion criteria were: (a) depression, (b) primary sleep disorders, (c) severe co-morbidity (i.e. Cumulative Illness Rating Scale item scores ≥ 3), (d) current pregnancy or having given birth in the past 3 months, (e) pharmacological treatment for fatigue that was started in the past 3 months, and (f) non-pharmacological therapies for fatigue that took place in the past 3 months. The full details of the enrolment criteria have been described previously^22^.

The medical ethics committee of the VU University Medical Center approved the TREFAMS-ACE programme (METc 2010/289; NL33451.029.10). Additionally, local feasibility statements were obtained from each participating medical centre. All study procedures performed were in accordance with the 1964 Helsinki Declaration and its later amendments or comparable ethical standards. Written informed consent was obtained from all individual participants included in the TREFAMS-ACE trials.

### Fatigue scales

In the TREFAMS-ACE studies, four self-reported fatigue questionnaires were used.

The *Checklist of Individual Strengths (CIS20r)* is a multidimensional questionnaire that consists of 20 items, divided into four fatigue dimensions and related behavioural aspects, including: (a) the subjective experience of fatigue (8 items), (b) reduction in motivation (4 items), (c) reduction of physical activity (3 items), and (d) reduction in concentration (5 items)^23,24^. The CIS20r focuses on fatigue in the past 2 weeks and for each item the person has to indicate on a 7-point scale to what extent the particular statement applies to her or him (yes, that is true – no, that is not true). However, information on the development phase of the CIS20r and the origin of the initial 24 items is missing^23,25^.

The *Modified Fatigue Impact Scale (MFIS)* is part of the Multiple Sclerosis Quality-of-Life Inventory (MSQLI), and assesses the perceived impact of fatigue on physical, cognitive, and psychosocial functioning^9,26^. From the questionnaire instructions it follows that “fatigue is a feeling of physical tiredness and lack of energy that many people experience from time to time”. Participants rate how often fatigue has affected 21 functions during the past 4 weeks using a 5-point scale (0 = never, 1 = rarely, 2 = sometimes, 3 = often, 4 = almost always). Besides a total MFIS score, subscale scores for physical (9 items), cognitive (10 items) and psychosocial fatigue (2 items) can be calculated^9,26^. The original 40-item Fatigue Impact Scale (FIS) was developed based on interviews of patients with MS about how fatigue affects their daily activities and social life^27^. The MFIS was derived from the FIS by a consensus meeting of an expert panel of the US National Multiple Sclerosis Society^9,26^.

The *Fatigue Severity Scale (FSS)* is an unidimensional questionnaire of 9 statements evaluating the severity but also the impact of fatigue in patients with MS. Items are scored from 1 to 7 (1 = completely disagree to 7 = completely agree)^28^. During development of the FSS, theoretical considerations and factor analysis were used to select the 9 items. Items were selected based on their ability to identify common features of fatigue in patients with MS and Systemic Lupus Erythematosus as compared to healthy controls^28^. Eight items ask about physical aspects of fatigue and the interference of fatigue in activities and responsibilities, and one item is related to cognitive aspect of fatigue (i.e. motivation is lower when fatigued). FSS scores are the mean (1–7) or the sum of the item scores (9–63), with lower scores indicating less fatigue.

The *SF36 vitality scale* is one of the subscales of the SF36, and consists of four items (i.e. feel full of life; having a lot of energy; feel worn out; feel tired), with a five-point frequency rating scale ranging from “all of the time” to “none of the time. The SF36 is a generic Quality of Life questionnaire, using a 4-week time frame. SF36 vitality yields a score on a 0–100 scale, with higher scores indicating more vitality^29^.

The MFIS and FSS are two well-known questionnaires that are used worldwide in MS rehabilitation research. The SF36 vitality subscale is usually not assessed independently, but as part of the (total) SF36. The multidimensional CIS20r is perhaps a quite new questionnaire in the field of MS. This valid and responsive questionnaire was the primary outcome in many RCTs on Cognitive Behavioural Therapy for fatigue in various diagnostic patient groups (e.g. Chronic Fatigue Syndrome, Diabetes, Cancer-related fatigue)^25^. The various time frames patients should have in mind when answering each set of fatigue questions differ, and were in this study not aligned to each other.

### Other measures

The baseline study questionnaire also included questions regarding socio-demographic characteristics (e.g. age, gender, level of education, employment status, living situation) and disease characteristics (e.g. date of diagnosis, date of first complaints, current type of MS). MS characteristics were also part of the enrolment examination by a rehabilitation physician.

### Statistical analysis

Together, the CIS20r, MFIS, FSS and SF36 vitality consist of nine fatigue subscale scores and 54 item scores. For each of the participants their baseline scores were used. All analyses were conducted using IBM SPSS 22.0 (Statistical Package for the Social Science). To study the distribution of scores, the floor and the ceiling effects of the nine subscales were studied by calculating the percentage of patients with the lowest or the highest scores on each subscale. At baseline, we expected no floor effects but possible ceiling effects (reversed for the SF36 vitality).

*Pearson correlation coefficients* between subscale scores were calculated within and between the four fatigue questionnaires. Multicollinearity (defined as a bivariate correlation > 0.70) was examined to ensure that the scales were not closely related.

#### Cluster analysis

There are various methodological approaches to identifying clinically important subgroups and one method is to identify clusters of characteristics that differentiate people using Cluster analysis^30,31^. Cluster analysis was applied using SPSS’s two-step clustering algorithm^31^. To facilitate interpretation of the results of the cluster analyses, the nine subscale scores were transformed into z-scores (mean 0, SD 1). Scores were checked for normal distribution. The clustering algorithm results in a number of clusters, and assigns patients to clusters so that the means for all variables are as different from each other as possible for the different clusters^30,31^. Such a clustering approach is useful in MS-related fatigue where there is known heterogeneity in perceived fatigue that might characterize patients with common fatigue profiles. In contrast to factor analytic approaches to test the scale constructs, cluster analysis is a person-centred technique^32^. The optimal number of clusters was automatically determined, using Schwarz’ Bayesian Information Criterion. As a measure of dissimilarity between groups the squared Euclidean distance between each pair of observations was used, where shorter distances indicated greater similarity. Ward’s hierarchical clustering method, which seeks to minimize the sum of squared errors between the groups regarding all variables, was used to verify the outcome of the two step clustering^30,31^. After evaluation of the data and the dendrogram plot, the groups were chosen based on the statistical significance of differences between the clusters. In order to validate the results obtained from the cluster analysis, an accurate discriminant function was estimated for classification of the MS patients in the fatigue profiles (clusters) formed. The classification accuracy of the estimated discriminant function was determined by the number of correctly classified individuals.

#### Factor analysis

In the next step, we tested if the 54 fatigue item scores behaved in accordance with a single, that is *unidimensional*, factor model, using factor analysis (principal axis factoring). The combined items were considered unidimensional if only one factor was extracted with an Eigenvalue equal to or greater than 1, with a percentage of explained variance of at least 50%^33^. Once unidimensionality was rejected, we used factor analysis to gain insights into the underlying dimensional structure of the combined 54 items, based on principal axis factoring with an oblimin rotation, permitting the underlying factors to be correlated.

## Results

### Patients

Table 1 shows the socio-demographic and disease-related characteristics of 264 participants with primary MS-related fatigue. The median duration of MS was 6.8 years, with a median Expanded Disability Status Scale (EDSS) disease severity score of 2.5 (range 0–6), a mean age of 48.1 years (range 19–68 yrs.) and 75% were women.

**Table 1:** Demographic and disease-related characteristics of the 264 patients with primary MS-related fatigue.

Demographic characteristic	Median/N	Range/%
Age (yrs)	48.1	19.6–68.1
Gender		
Male	66	25
Female	198	75
Level of Education		
Low	17	6.4
Intermediate	122	46.2
High	125	47.3
Disability pension		
No	113	42.8
Yes, partial	50	18.9
Yes, full	101	38.3
Employment^*a*^		
Full-time	28	10.7
Part-time	99	37.9
Not	119	45.6
(Early) retirement	10	3.8
Student	5	1.9
Living arrangement		
Living with partner	204	77.3
Living alone/with children/other	60	22.7
**Disease characteristics**		
Type of MS		
Relapsing-Remitting	190	72
Primary Progressive	24	9.1
Secondary Progressive	32	12.1
Unknown	18	6.9
Duration of MS (yrs)	6.8	0.14–30.7
EDSS	2.5	0.0–6.0
Member of a patient organisation^*a*^		
Yes	119	45.4
No	143	54.6

^*a*^Due to missing values the total number is not equal to 264. Abbreviations: EDSS – Expanded Disability Status Scale, MS – Multiple Sclerosis, yrs - years.

### Distribution of fatigue scores

Mean scores on all fatigue subscales, as well as the percentage floor and ceiling effects are shown in Table 2. Overall, floor and ceiling effects were small (<5%). However, against expectations, two of the three MFIS subscales and three of the four CIS20r subscales showed some floor effects in this study group of severely fatigued patients. Some participants reported maximum fatigue scores on the CIS20r subscales, the FSS, and MFIS psychosocial. Participants had mean fatigue scores of 5.3 on the FSS (SD 0.9), 92.0 (SD 17.2) on the CIS20r total, 44.2 (SD 12.3) on the MFIS total and 41.9 (SD 14.2) on the SF36 vitality.

**Table 2:** Scores on the fatigue subscales.

Measure	Items	Theoretical Range	Mean (sd)	Floor Effect %	Ceiling Effect %
CIS20r total	20	20–140	92.0 (17.2)	0	0
CIS20r fatigue	8	8–56	43.3 (7.7)	0	1.9
CIS20r concentration	5	5–35	20.9 (7.6)	2.3	2.3
CIS20r motivation	4	4–28	15.1 (5.4)	1.5	0.8
CIS20r phys. activities	3	3–21	12.8 (4.8)	2.3	4.9
MFIS total	21	0–84	44.2 (12.3)	0	0
MFIS physical	9	0–36	21.0 (5.4)	0	0
MFIS cognitive	10	0–40	19.2 (7.8)	0.4	0
MFIS psychosocial	2	0–8	4.0 (1.7)	4.5	1.1
FSS	9	1–7	5.3 (0.9)	0	0.4
SF36 vitality	4	0–100	41.9 (14.2)	0	0

Abbreviations: CIS20r - Checklist of Individual Strength, FSS - Fatigue Severity Scale, MFIS - Modified Fatigue Impact Scale, sd – standard deviation, SF36 vitality – Short Form 36 vitality.

### Correlation of fatigue subscales

Table 3 shows the correlation coefficients within and between fatigue scales. In general, most correlations were low, indicating that despite some overlap the subscales were measuring different constructs and illustrate the multidimensional character of MS-related fatigue. The low correlation of the FSS with nearly all other subscales provides evidence that the FSS is measuring a different aspect of fatigue. The subscale correlations within the CIS20r are lower than the correlations within the MFIS. This table also shows that the MFIS cognitive subscale and the CIS20r concentration subscale are highly correlated (r = 0.799), seemingly measuring the same construct.

**Table 3:** Pearson correlation coefficients of fatigue subscales in 264 patients with MS.

	CIS20r concentration	CIS20r motivation	CIS20r physical activities	MFIS physical	MFIS cognitive	MFIS psycho-social	FSS	SF36 vitality
CIS20r fatigue	0.219	0.293	0.340	0.499	0.198	0.431	0.403	−0.640
CIS20r concentration		0.227	0.247	0.210	0.799	0.282	0.243	−0.290
CIS20r motivation			0.320	0.244	0.250	0.344	0.201	−0.355
CIS20r phys. activities				0.387	0.292	0.340	0.289	−0.357
MFIS physical					0.423	0.657	0.383	−0.393
MFIS cognitive						0.436	0.271	−0.357
MFIS psychosocial							0.436	−0.446
FSS								−0.355

Abbreviations: CIS20r - Checklist of Individual Strength, FSS - Fatigue Severity Scale, MFIS - Modified Fatigue Impact Scale, sd – standard deviation,

SF36 vitality – Short Form 36 vitality. The direction of the SF36 vitality is opposite to the direction of the other scales, resulting in negative coefficients.

All correlations were significant at the 0.01 level (2-tailed).

### Fatigue profiles

The cluster analysis was based on complete data from 262 patients and initially resulted in a two-profile solution. The CIS20r concentration was excluded from the cluster analysis due to multicollinearity of the subscales CIS20r concentration and MFIS cognitive. The first fatigue profile included 88 patients with relatively low average scores on all subscales, whereas patients with the other profile had relatively high scores on all subscales (Table 4a). At the expense of losing some accuracy in patient classification, we also investigate a three-cluster solution. From the large cluster (174 patients) another profile was extracted that included fatigued patients with average scores (z-scores close to 0) who were therefore less troubled by cognitive fatigue, vitality, and physical activities compared to members of the third profile who showed high scores on all subscales (Table 4b). This three-profile solution correctly classified 86.3% of the patients, whereas the two profiles correctly classified 96.6% of the patients (Table 4).

**Table 4:** Cluster analyses resulted in two (A) or three fatigue profiles (B).

A. Two fatigue profile solution			
Z-scores	Profile 1 n = 88	Profile 2 + 3 n = 174	
CIS20r fatigue	−0.73	0.37	
CIS20r motivation	−0.37	0.19	
CIS20r phys. activities	−0.48	0.25	
MFIS physical	−0.96	0.48	
MFIS cognitive	−0.56	0.28	
MFIS psychosocial	−1.00	0.50	
FSS	−0.78	0.40	
SF36 vitality	0.71	−0.37	
**B. Three fatigue profile solution**			
**Z-scores**	**Profile 1 n = 88**	**Profile 2 n = 96**	**Profile 3 n = 78**
CIS20r fatigue	−0.73	0.22	0.57
CIS20r motivation	−0.37	−0.39	0.90
CIS20r phys. activities	−0.48	−0.04	0.59
MFIS physical	−0.96	0.31	0.69
MFIS cognitive	−0.56	0.05	0.57
MFIS psychosocial	−1.00	0.36	0.68
FSS	−0.78	0.47	0.32
SF36 vitality	0.71	−0.10	−0.70

(A) 96.6% of the patients were correctly classified.

(B) 86.3% of the patients were correctly classified.

These fatigue profiles could not be intuitively linked to the clinically expected dimensions of MS-related fatigue, which includes physical, cognitive or mental fatigue, or the psychosocial or distress dimension (with the subscales reduced motivation and reduced activities). The distinction between these profiles was due to the severity of the fatigue, evidenced by the high vs. low z-scores on all subscales.

### Unidimensionality

Factor analysis of all 54 items showed that there was no unidimensionality among the 54 items. Eight factors with Eigenvalues equal to or greater than 1.0 were extracted, which together explained 57.1% of the total variance (Table 5). The first factor with a loading of 11/54 items explained only 31% of the total variance. The results of the item-to-factor loadings are presented in Table 6. A minimum item-factor loading of 0.500 was found in 39/54 items. In nine items, all with low factor loadings, the difference in loadings on several factors was small (<0.200), meaning that these items could not be uniquely assigned to one of the eight factors. Table 6 shows that the items of subscales of the CIS20r, MFIS, and the FSS almost all load on their own factor (e.g. CIS20r fatigue items load on factor 1, the highly correlated CIS20r concentration items and MFIS cognitive items load on factor 2, MFIS physical items load on factor 3, CIS20r motivation load on factor 4, FSS items load on factor 5 and 7, CIS20r physical activities load on factor 6, and factor 8 contains one CIS20r fatigue item that also loads on factor (1). The eight factors almost confirm the original factor structure of the subscales of the 4 included fatigue questionnaires.

**Table 5:** Results of the Factor Analysis of 54 Fatigue Questions from four Fatigue Questionnaires.

Total Variance Explained							
Factor	Initial Eigenvalues			Extraction Sums of Squared Loadings			Rotation Sums of Squared Loadings^a^
	Total	% of Variance	Cumulative %	Total	% of Variance	Cumulative %	Total
1	17.144	31.749	31.749	16.743	31.006	31.006	8.804
2	5.946	11.012	42.760	5.561	10.297	41.304	11.960
3	2.872	5.319	48.079	2.440	4.519	45.822	7.510
4	2.399	4.443	52.523	1.955	3.620	49.442	6.304
5	1.888	3.497	56.019	1.438	2.664	52.105	5.578
6	1.587	2.938	58.958	1.185	2.195	54.300	7.554
7	1.294	2.396	61.354	0.801	1.484	55.784	2.932
8	1.171	2.169	63.523	0.727	1.347	57.131	3.525
9	0.962	1.782	65.305				
10^b^	0.937	1.736	67.041				

^a^When factors are correlated, sums of squared loadings cannot be added to obtain a total variance.

^b^Table has been shortened after 10 of 54 factors. Extraction Method: Principal Axis Factoring. Rotation Method: Oblimin with Kaiser Normalization.

**Table 6:** Pattern Matrix of Factor Analysis of 54 fatigue items from the CIS20r, FSS, SF36 vitality and MFIS.

Fatigue Item	Factor							
	1	2	3	4	5	6	7	8
CIS20r_1c_fatigue	**0.765**							
CIS20r_2_motivation	0.180			**0.593**		0.186		
CIS20r_3c_conc	0.132	**0.802**	−0.137					
CIS20r_4c_fatigue	**0.642**						−0.102	0.321
CIS20r_5_motivation				**0.740**				
CIS20r_6_fatigue	**0.578**			0.235				
CIS20r_7_act						**0.839**		−0.109
CIS20r_8_conc		0.778	−0.180			0.119		
CIS20r_9c_fatigue	0.227							**0.549**
CIS20r_10c_act						**0.858**		
CIS20r_11_conc		**0.841**	−0.236					
CIS20r_12_fatigue	**0.678**			0.109				
CIS20r_13c_conc		**0.730**	−0.218				0.100	
CIS20r_14c_fatigue*	***0.329***		*0.119*	*0.125*		*0.121*		*0.296*
CIS20r_15_motivation				**0.810**			0.125	
CIS20r_16c_fatigue	**0.643**					0.108	0.138	
CIS20r_17c_act		0.139				**0.619**		
CIS20r_18c_motivation				**0.575**				0.150
CIS20r_19c_conc		**0.742**	−0.187					0.178
CIS20r_20_fatigue	***0.454***		*0.112*	*0.186*			*0.170*	*0.144*
SF36_23c				**−0.508**	−0.188			
SF36_27c*	***−0.417***			−0.306	−0.126	−0.107		
SF36_29	−0.541		−0.155		−0.108			−0.179
SF36_31	−0.746		−0.112					
FSS_1		*0.148*		*0.147*	***0.419***			
FSS_2		*−0.111*					***0.358***	*0.117*
FSS_3*	***0.468***				*0.203*		*0.326*	*−0.102*
FSS_4*					*0.432*		***0.438***	
FSS_5					***0.492***		*0.220*	*0.248*
FSS_6			0.169		0.150		0.644	−0.116
FSS_7		*0.103*			***0.493***	*0.256*	*0.275*	
FSS_8*	*0.344*				***0.448***			*−0.158*
FSS_9	0.160				**0.617**	0.144		
MFIS_1_cognitive	0.131	**0.573**	0.228		0.136		−0.139	
MFIS_2_cognitive	0.134	**0.758**	0.151					−0.120
MFIS_3_cognitive		**0.739**	0.116					
MFIS_4_fys*		**0.361**	0.338				0.159	0.106
MFIS_5_cognitive		**0.695**	0.170					
MFIS_6_fys		0.118	**0.567**		−0.104	0.129	0.218	
MFIS_7_fys			**0.542**	0.201	0.128		−0.155	
MFIS_8_psychosoc*		*0.134*	***0.383***	*0.218*	*0.318*		*−0.245*	
MFIS_9_psychosoc			**0.505**	0.105	0.147	0.160		
MFIS_10_fys	0.118		**0.580**			0.135	0.306	
MFIS_11_cognitive	−0.127	**0.566**	0.177		0.136		−0.108	
MFIS_12_cognitive		**0.649**	0.169		0.124		−0.150	
MFIS_13_fys*			***0.398***				*0.180*	*0.387*
MFIS_14_fys	0.247		**0.457**					0.220
MFIS_15_cognitive		**0.761**	0.166					
MFIS_16_cognitive		**0.769**	0.150					
MFIS_17_fys		0.117	**0.595**			0.144	0.183	
MFIS_18_cognitive		**0.767**	0.153					
MFIS_19_cognitive		**0.869**						−0.112
MFIS_20_fys			**0.667**			0.158	0.107	
MFIS_21_fys*	*0.300*		***0.465***	*−0.108*		*0.121*		

Extraction Method: Principal Axis Factoring, Rotation Method: Oblimin with Kaiser Normalization, Rotation converged in 25 iterations.

Small item-to-factor loadings < −0.100–0.100 > are not presented.

For each item the highest factor loading **in bold**. Fatigue items marked with an asterix do not discriminate enough and are actually redundant. In Italic: items with loading difference  < 0.200.

## Discussion

This study aimed to discover subgroups of MS patients with comparable fatigue complaints, and resulted in the identification of three homogeneous profiles of primary MS-related fatigue. However, the identified fatigue profiles did not show that existing fatigue domains were decisive in the formation of the clusters. Fatigue profiles mainly distinguished patients based on the severity of their score on all domains. The multiple dimensions of fatigue had no influence on cluster formation. Therefore, in patients with a treatment indication due to severe primary MS-related fatigue, the fatigue profile solutions are not helpful in more appropriate targeting of the optimal fatigue intervention. Also in other diagnostic groups (i.e. Parkinson’s Disease, Colorectal Cancer), patient profiling did not result in clear differentiation based on the most affected fatigue domains^34,35^. An explanation for the absence of distinct profiles for type of fatigue might be related to the inability of patients to make further distinctions when fatigue becomes very severe. This fits with the theoretical model of low energy in chronically ill patients described by Lerdal^36^. The Unidimensional Fatigue Impact Scale (U-FIS) developed in MS patients showed from the item hierarchy found that the physical aspects of fatigue represent mild levels of the functional impact of fatigue, cognitive aspects capture moderate levels of fatigue impact and emotional aspects represent the most severe levels of fatigue impact^37^.

Although the results of the cluster analysis suggested that fatigue may be an unidimensional construct, the factor analysis clearly showed that an unidimensional fatigue scale could not be confirmed. Factor analysis showed evidence for eight fatigue factors, comparable to most of the subscales of the questionnaires used in this analysis. This endorses the multidimensional nature of fatigue.

The dimensionality of the CIS20r and MFIS have already been repeatedly investigated by others, mostly confirming the multidimensionality of both scales^25,38–40^. The MFIS is commonly used to generate an overall score of fatigue. However, its 21 items do not fit the unidimensional Rasch model mainly because of multidimensionality^41,42^. Valid physical and cognitive subscales of the MFIS were derived after deletion of some items^41^. In their paper, Lundgren-Nilsson *et al*. present a 15- and a 6-item Rasch transformed interval fatigue scale fulfilling unidimensionality and a transformation table to adjust scores derived from the original MFIS^42^. For the FSS, item reductions have been proposed in response to non-fitting items^43,44^.

Recently, some unidimensional fatigue scales were developed in which fatigue is actually considered a continuum^45^. The PROMIS fatigue item bank (PROMIS stands for Patient Reported Outcomes Measurement Information System) consists of 95 items (including 4 items of the SF36 vitality) and measures the experience of fatigue and its impact on daily functioning^46^. With regard to cancer-related fatigue, the EORTC fatigue item bank (44 items) specifically aims at measuring general and physical fatigue^47^. Another cancer-related fatigue bank of 72 items, covering various aspects of fatigue, explained 57.5% of the fatigue variance with one factor^48^. Fatigue item banks are useful in computer adaptive testing. However, it should be noted that due to strict requirements of the Rasch model or Item Response Theory models, measurement rigor and assumptions, the fatigue construct of the remaining items and short forms (e.g. PROMIS-Fatigue(MS)) is increasingly fading^40,49–51^. In contrast to these unidimensional fatigue scales and item banks, construct validation of fatigue in rheumatoid arthritis resulted in a multidimensional computerized adaptive test^52^.

De Raaf *et al*.^53^ considered three options for the nature of fatigue: the unidimensional concept, the multidimensional concept, and the multiple symptoms concept. In the *multiple symptoms concept of fatigue* these authors proposed that fatigue might not be one, albeit multidimensional, symptom or concept, but an expression of separate symptoms that are collectively called fatigue. Each symptom represents an unidimensional scale and scores of the different symptoms cannot be summed. Supportive of the multiple symptoms concept of fatigue, de Raaf *et al*.^53^ argued that (1) symptoms respond differently to their intensity in cross-sectional studies comparing various groups of patients, (2) the course of the intensity of symptoms is different in longitudinal follow-up studies, (3) symptoms correlate differently with other variables, and (4) symptoms behave differently in their response to interventions aimed at relieving fatigue. Furthermore, the pathogenesis of symptoms may differ, and symptoms show low mutual correlation. The low mutual correlations of the CIS20r subscales and the MFIS subscales found in our study (Table 3) might be indications for the multiple symptoms concept of fatigue.

The following study limitations should be considered. The study population purposely consisted of severely fatigued patients with MS. The CIS20r fatigue subscale was used to screen trial participants. We suspect that using for instance the FSS or the MFIS as screening instrument, would have led to small changes in the composition of the study population, with some participants not being included, and others being included. Scores on all four fatigue questionnaires were not available for non-fatigued patients, and those patients that were excluded during the screening process. However, the aim of our study was not to discriminate non-fatigued patients from fatigued patients, but primarily to profiling severely fatigued patients in order to specify their treatment and improve treatment efficiency. We assume that the inclusion of non-fatigue MS patients may have even strengthened the profiles based on the severity scores. Furthermore, we used cluster analysis and factor analysis on the same dataset, which might seem unusual at first glance^32^. Cluster analysis of fatigue subscale scores aimed to identify particular clusters of individuals showing the same fatigue pattern, whereas factor analysis of the fatigue items was applied to study the dimensionality of fatigue and the type of fatigue items that grouped together. The FSS and the SF36 vitality were considered unidimensional. The CIS20r and the MFIS were constructed as multidimensional scales, although the use of total CIS20r and MFIS scores is still commonplace. Based on the outcome of the cluster analysis and in view of the constructs of the four fatigue questionnaires, we nevertheless decided to investigate the factor structure of the fatigue items under the assumption that a unidimensional scale could be distinguished.

Almost 90% of the participants (n = 234) preferred to complete the internet questionnaire. Due to this large imbalance with those participants who wanted to complete the paper version, we did not perform sensitivity analyses to investigate whether paper and internet assessments led to different clusters or factors.

Generalizability of the findings should be related to our aim and study population.

## Conclusions

Fatigue related to Multiple Sclerosis (MS) is considered a multidimensional symptom, manifesting in several dimensions such as physical, cognitive, and psychosocial fatigue. The present cross-sectional study applied cluster analysis to the nine fatigue subscales with the objective of identifying distinct fatigue profiles that might require different rehabilitation treatment approaches in patients with primary MS-related fatigue. The fatigue profiles discovered did not differentiate between clinically expected dimensions of fatigue. One unidimensional fatigue construct within the 54 fatigue questions could not be confirmed, and the four fatigue instruments CIS20r, MFIS, FSS and SF36 vitality appear to measure different fatigue constructs.

The results of this study imply that for diagnosis and initiation of fatigue treatment, one simple fatigue (sub-)scale, such as the CIS20r fatigue, is sufficient. No currently available study has shown that dimension-specific treatment of fatigue leads to better outcomes. In terms of clinical practice this means that there is as yet no reason to measure multidimensional aspects of fatigue in primary fatigued patients with MS.

## Supplementary information

Supplementary Files.

## Data Availability

The datasets used and/or analysed during the current study are available from the corresponding author on reasonable request.
