# Supplementary material for: Fatigue Profiles in Patients with Multiple Sclerosis are Based on Severity of Fatigue and not on Dimensions of Fatigue
Source: Sci Rep. 2020 Mar 5;10:4167. doi: 10.1038/s41598-020-61076-1 (PMC7058058; doi:10.1038/s41598-020-61076-1)
Supplement: Supplementary file 1 — Supplementary Files. [file 41598_2020_61076_MOESM1_ESM.docx]

**Supplementary File 1.**

**REFERENCES ABOUT DEFINITIONS OF FATIGUE**

**FATIGUE PROFILES IN PATIENTS WITH MULTIPLE SCLEROSIS ARE BASED ON SEVERITY OF FATIGUE AND NOT ON DIMENSIONS OF FATIGUE**

Heleen Beckerman PhD^1,2,3^*, Isaline CJM Eijssen PhD OT^1,2,3^, Jetty van Meeteren PhD MD^4^, Marion C Verhulsdonck MD^5^, Vincent de Groot, PhD MD^1,2,3^, and the TREFAMS-ACE study group

1. Department of Rehabilitation Medicine, Amsterdam University Medical Centers, Vrije Universiteit, Amsterdam, The Netherlands; 2. Amsterdam Public Health research institute, Amsterdam University Medical Centers, The Netherlands; 3. MS Center Amsterdam, Amsterdam University Medical Centers, The Netherlands; 4. Rijndam Rehabilitation, location Erasmus MC University Medical Center Rotterdam, The Netherlands; 5. Rehabilitation Center St Maartenskliniek Nijmegen, The Netherlands

*E-mail corresponding authors: [h.beckerman@amsterdamumc.nl](mailto:h.beckerman@amsterdamumc.nl)

**References about definitions of fatigue**

*(without pretending to be complete)*

Aaronson LS, Teel CS, Cassmeyer V, Neuberger GB, Pallikkathayil L, Pierce J, Press AN, Williams PD, Wingate A. Defining and measuring fatigue. Image J Nurs Sch. 1999;31(1):45-50.

Barofsky I, Legro MW. Definition and measurement of fatigue. Rev Infect Dis. 1991 Jan-Feb;13 Suppl 1:S94-7.

Barsevick AM, Cleeland CS, Manning DC, O'Mara AM, Reeve BB, Scott JA, Sloan JA; ASCPRO (Assessing Symptoms of Cancer Using Patient-Reported Outcomes). ASCPRO recommendations for the assessment of fatigue as an outcome in clinical trials. J Pain Symptom Manage. 2010 Jun;39(6):1086-99.

Berger AM, Mooney K, Alvarez-Perez A, Breitbart WS, Carpenter KM, Cella D, Cleeland C, Dotan E, Eisenberger MA, Escalante CP, Jacobsen PB, Jankowski C, LeBlanc T, Ligibel JA, Loggers ET, Mandrell B, Murphy BA, Palesh O, Pirl WF, Plaxe SC, Riba MB, Rugo HS, Salvador C, Wagner LI, Wagner-Johnston ND, Zachariah FJ, Bergman MA, Smith C; National comprehensive cancer network. Cancer-Related Fatigue, Version 2.2015. J Natl Compr Canc Netw. 2015 Aug;13(8):1012-39.

Beurskens AJ, Bultmann U, Kant I, Vercoulen JH, Bleijenberg G, Swaen GM. Fatigue among working people: validity of a questionnaire measure. Occup Environ Med 2000; 57(5):353-357.

Brown RG, Dittner A, Findley L, Wessely SC. The Parkinson fatigue scale. Parkinsonism Relat Disord 2005;11(1):49e55.

Chaudhuri A, Behan PO. Fatigue in neurological disorders. Lancet 2004,363:978-988.

DeLuca J. Fatigue: its definition, its study, and its future. In: Fatigue as a window to the brain. Edited by DeLuca J. Cambridge: The MIT Press; 2005.

Dittner AJ, Wessely SC, Brown RG. The assessment of fatigue: a practical guide for clinicians and researchers. J Psychosom Res. 2004;56(2):157–170.

Doward LC, Meads DM, Fisk J, Twiss J, Hagell P, Oprandi NC, Grand'Maison F, Bhan V, Arbizu T, Kohlmann T, Brassat D, Eckert BJ, McKenna SP. International development of the Unidimensional Fatigue Impact Scale (U-FIS). Value Health. 2010 Jun-Jul;13(4):463-8.

Enoka RM, Duchateau J. Translating Fatigue to Human Performance. Med Sci Sports Exerc 2016; 48(11):2228-2238.

Eshragh J, Dhruva A, Paul SM, Cooper BA, Mastick J, Hamolsky D, Levine JD, Miaskowski C, Kober KM. Associations Between Neurotransmitter Genes and Fatigue and Energy Levels in Women After Breast Cancer Surgery. J Pain Symptom Manage 2017;53(1):67-84.e7.

Finsterer J, Mahjoub SZ. Fatigue in healthy and diseased individuals. Am J Hosp Palliat Care. 2014 Aug;31(5):562-75.

Fisk JD, Pontefract A, Ritvo PG, Archibald CJ, Murray TJ. The impact of fatigue on patients with multiple sclerosis. Can J Neurol Sci. 1994 Feb;21(1):9-14.

Ford H, Trigwell P, Johnson M. The nature of fatigue in multiple sclerosis. J Psychosom Res. 1998 Jul;45(1):33-8.

Hensyl WR (ed), Stedman’s Medical Dictionary, 25^th^ edn, Williams & Wilkins, Baltimore, MD, 1990.

Heuchert JP, McNair DM. Profile of mood states 2nd edition (POMS 2). North Tonawanda, NY: Multi-Health Systems Inc.; 2012.

International Classification of Diseases, Tenth Revision, Clinical Modification (ICD10-CM)]. Available from: <http://www.cdc.gov/nchs/icd/icd10cm.htm>

Jason LA, Evans M, Brown M, Porter N. What is fatigue? Pathological and non-pathological fatigue. PM R. 2010 May;2(5):327-31.

Jhamb M, Weisbord SD, Steel JL, Unruh M. Fatigue in patients receiving maintenance dialysis: a review of definitions, measures, and contributing factors. Am J Kidney Dis. 2008 Aug;52(2):353-65.

Kennedy HG. Fatigue and fatigability. Br J Psychiatry. 1988 Jul;153:1-5.

Kluger BM, Krupp LB, Enoka RM. Fatigue and fatigability in neurologic illnesses: proposal for a unified taxonomy. Neurology 2013;80:409–16.

Kluger BM, Herlofson K, Chou KL, Lou JS, Goetz CG, Lang AE, Weintraub D, Friedman J. Parkinson's disease-related fatigue: A case definition and recommendations for clinical research. Mov Disord. 2016 May;31(5):625-31.

Lerdal A. A concept analysis of energy: Its meaning in the lives of three individuals with chronic illness. Scand J Caring Sci 1998;12:3–10.

Lerdal A. A theoretical extension of the concept of energy through an empirical study. Scand J Caring Sci. 2002 Jun;16(2):197-206.

Lewis G, Wessely S. The epidemiology of fatigue: more questions than answers. J Epidemiol Community Health. 1992 Apr;46(2):92-7.

Loy BD, Cameron MH, O'Connor PJ. Perceived fatigue and energy are independent unipolar states: Supporting evidence. Med Hypotheses. 2018 Apr;113:46-51.

MedlinePlus Medical Encyclopedia-Fatigue: U.S. National Library of Medicine. Available from: <https://medlineplus.gov/ency/article/003088.htm>.

Mills RJ, Young CA. A medical definition of fatigue in multiple sclerosis. QJM 2008; 101: 49–60.

Multiple Sclerosis Council for Clinical Practice Guidelines. Fatigue and multiple sclerosis: evidence-based management strategies for fatigue in multiple sclerosis. Washington (DC): Paralyzed Veterans of America; 1998.

NCI Dictionary of Cancer Terms National Cancer Institute. Available from: <https://http://www.cancer.gov/publications/dictionaries/cancerterms/def/fatigue>.

Phillips RO. A review of definitions of fatigue – And a step towards a whole definition. Transportation Research Part F: Traffic Psychology and Behaviour 2015; 29: 48-56.

Ream E, Richardson A. Fatigue: a concept analysis. Int J Nurs Stud. 1996 Oct;33(5):519-29.

Riley WT, Rothrock N, Bruce B, Christodolou C, Cook K, Hahn EA, Cella D. Patient-reported outcomes measurement information system (PROMIS) domain names and definitions revisions: further evaluation of content validity in IRT-derived item banks. Qual Life Res. 2010 Nov;19(9):1311-21.

Shen J, Barbera J, Shapiro CM. Distinguishing sleepiness and fatigue: focus on definition and measurement. Sleep Med Rev. 2006 Feb;10(1):63-76.

Tiesinga LJ, Dassen TW, Halfens RJ. Fatigue: a summary of the definitions, dimensions, and indicators. Nurs Diagn 1996;7(2):51-62.

Trendall J. Concept analysis: chronic fatigue. J Adv Nurs 2000;32(5):1126-31.

WebMD Fatigue Directory. Available from:

<http://www.webmd.com/sleep-disorders/fatigue-directory>

**Supplementary File 2.**

**SYSTEMATIC REVIEWS OF FATIGUE QUESTIONNAIRES**

**FATIGUE PROFILES IN PATIENTS WITH MULTIPLE SCLEROSIS ARE BASED ON SEVERITY OF FATIGUE AND NOT ON DIMENSIONS OF FATIGUE**

Heleen Beckerman PhD^1,2,3^*, Isaline CJM Eijssen PhD OT^1,2,3^, Jetty van Meeteren PhD MD^4^, Marion C Verhulsdonck MD^5^, Vincent de Groot, PhD MD^1,2,3^, and the TREFAMS-ACE study group

1. Department of Rehabilitation Medicine, Amsterdam University Medical Centers, Vrije Universiteit, Amsterdam, The Netherlands; 2. Amsterdam Public Health research institute, Amsterdam University Medical Centers, The Netherlands; 3. MS Center Amsterdam, Amsterdam University Medical Centers, The Netherlands; 4. Rijndam Rehabilitation, location Erasmus MC University Medical Center Rotterdam, The Netherlands; 5. Rehabilitation Center St Maartenskliniek Nijmegen, The Netherlands

*E-mail corresponding authors: [h.beckerman@amsterdamumc.nl](mailto:h.beckerman@amsterdamumc.nl)

**Systematic Reviews of Fatigue Questionnaires**
*(without pretending to be complete)*

Ad Hoc Committee on Systemic Lupus Erythematosus Response Criteria for Fatigue. Measurement of fatigue in systemic lupus erythematosus: a systematic review. Arthritis Rheum. 2007;57(8):1348-57.

Agasi-Idenburg C, Velthuis M, Wittink H. Quality criteria and user-friendliness in self-reported questionnaires on cancer-related fatigue: a review. J Clin Epidemiol 2010;63(7):705-11.

Debouverie M, Pittion-Vouyovitch S, Guillemin F. [Reconsidering fatigue at the onset of multiple sclerosis]. Rev Neurol (Paris). 2009 Mar;165 Suppl 4:S135-44.

Dittner AJ, Wessely SC, Brown RG. The assessment of fatigue: a practical guide for clinicians and researchers. J Psychosom Res. 2004 Feb;56(2):157-70.

Egerton T, Riphagen II, Nygård AJ, Thingstad P, Helbostad JL. Systematic content evaluation and review of measurement properties of questionnaires for measuring self-reported fatigue among older people. Qual Life Res. 2015 Sep;24(9):2239-55.

Elbers RG, Rietberg MB, van Wegen EE, Verhoef J, Kramer SF, Terwee CB, Kwakkel G. Self-report fatigue questionnaires in multiple sclerosis, Parkinson's disease and stroke: a systematic review of measurement properties. Qual Life Res. 2012 Aug;21(6):925-44.

Friedman JH, Alves G, Hagell P, Marinus J, Marsh L, Martinez-Martin P, Goetz CG, Poewe W, Rascol O, Sampaio C, Stebbins G, Schrag A. Fatigue rating scales critique and recommendations by the Movement Disorders Society task force on rating scales for Parkinson's disease. Mov Disord. 2010; 25(7): 805-22.

Féasson L, Camdessanché JP, El Mandhi L, Calmels P, Millet GY. Fatigue and neuromuscular diseases. Ann Readapt Med Phys. 2006 Jul;49(6):289-300, 375-84.

Hewlett S, Hehir M, Kirwan JR. Measuring fatigue in rheumatoid arthritis: a systematic review of scales in use. Arthritis Rheum. 2007 Apr 15;57(3):429-39.

Hewlett S, Dures E, Almeida C. Measures of fatigue: Bristol Rheumatoid Arthritis Fatigue Multi-Dimensional Questionnaire (BRAF MDQ), Bristol Rheumatoid Arthritis Fatigue Numerical Rating Scales (BRAF NRS) for severity, effect, and coping, Chalder Fatigue Questionnaire (CFQ), Checklist Individual Strength (CIS20R and CIS8R), Fatigue Severity Scale (FSS), Functional Assessment Chronic Illness Therapy (Fatigue) (FACIT-F), Multi-Dimensional Assessment of Fatigue (MAF), Multi-Dimensional Fatigue Inventory (MFI), Pediatric Quality Of Life (PedsQL) Multi-Dimensional Fatigue Scale, Profile of Fatigue (ProF), Short Form 36 Vitality Subscale (SF-36 VT), and Visual Analog Scales (VAS). Arthritis Care Res (Hoboken). 2011 Nov;63 Suppl 11:S263-86.

Hjollund NH, Andersen JH, Bech P. Assessment of fatigue in chronic disease: a bibliographic study of fatigue measurement scales. Health Qual Life Outcomes 2007;5:12.

Johnson SL. The concept of fatigue in multiple sclerosis. J Neurosci Nurs 2008;40(2):72-7.

Ju A, Unruh ML, Davison SN, Dapueto J, Dew MA, Fluck R, Germain M, Jassal SV, Obrador G, O'Donoghue D, Tugwell P, Craig JC, Ralph AF, Howell M, Tong A. Patient-Reported Outcome Measures for Fatigue in Patients on Hemodialysis: A Systematic Review. Am J Kidney Dis. 2018 Mar;71(3):327-343.

Kos D, Kerckhofs E, Ketelaer P, Duportail M, Nagels G, D’Hooghe MB et al . Self-report assessment of fatigue in multiple sclerosis: a critical evaluation. Occup Ther Health Care 2003;17:45-62.

Kos D, Kerckhofs E, Ketelaer P, Duportail M, Nagels G, D'Hooghe M, Nuyens G. Self-report assessment of fatigue in multiple sclerosis: a critical evaluation. Occup Ther Health Care 2004;17(3-4): 45-62.

Minton O, Stone P. A systematic review of the scales used for the measurement of cancer-related fatigue (CRF). Ann Oncol. 2009 Jan;20(1):17-25.

Seyidova-Khoshknabi D, Davis MP, Walsh D. Review article: a systematic review of cancer-related fatigue measurement questionnaires. Am J Hosp Palliat Care. 2011 Mar;28(2):119-29.

Swain MG. Fatigue in chronic disease. Clin Sci (Lond) 2000;99(1):1-8.

Tyson SF, Brown P. How to measure fatigue in neurological conditions? A systematic review of psychometric properties and clinical utility of measures used so far. Clin Rehabil. 2014;28(8):804-816.

Whitehead L. The measurement of fatigue in chronic illness: a systematic review of unidimensional and multidimensional fatigue measures. J Pain Symptom Manage 2009;37(1):107–128.
